# Are hiking recommendations one-size-fits-all? Insights into cardiovascular safety and trail demands

**DOI:** 10.1093/bmb/ldaf019

**Published:** 2025-11-19

**Authors:** Marco Vecchiato, Nicola Borasio, Emiliano Scettri, Dario Cangialosi, Stefano Palermi, Sandro Savino, Andrea Ermolao, Daniel Neunhaeuserer

**Affiliations:** Sports and Exercise Medicine Division, Department of Medicine, University of Padova, Via Giustiniani 2, 35128, Padova, Italy; Sports and Exercise Medicine Division, Department of Medicine, University of Padova, Via Giustiniani 2, 35128, Padova, Italy; Institute of Mountain Emergency Medicine, EURAC Research, Via Ipazia 2, 39100, Bolzano, Italy; Sports and Exercise Medicine Division, Department of Medicine, University of Padova, Via Giustiniani 2, 35128, Padova, Italy; Sports and Exercise Medicine Division, Department of Medicine, University of Padova, Via Giustiniani 2, 35128, Padova, Italy; Department of Medicine and Surgery, UniCamillus-Saint Camillus International University of Health Sciences, Via di Sant’Alessandro 8, 00187, Rome, Italy; Department of Medicine, University of Padova, Via Giustiniani 2, 35128, Padova, Italy; Sports and Exercise Medicine Division, Department of Medicine, University of Padova, Via Giustiniani 2, 35128, Padova, Italy; Sports and Exercise Medicine Division, Department of Medicine, University of Padova, Via Giustiniani 2, 35128, Padova, Italy

**Keywords:** hiking, exercise intensity, excursion, exercise prescription, mountain, cardiopulmonary exercise testing

## Abstract

**Introduction:**

Hiking is an outdoor activity with not only significant health benefits but also associated risks, especially for individuals with cardiovascular conditions. Current trail recommendations lack personalization, potentially increasing the risk of adverse events during hiking.

**Sources of data:**

Prospective, cross-sectional study combining outpatient cardiopulmonary exercise testing with monitored outdoor hiking. Data were collected via portable gas analysis, heart rate monitors, and an official meteorological station.

**Areas of agreement:**

Hiking intensity and cardiorespiratory responses vary widely. Cardiovascular risk and trail slope were found to influence the exertion required to complete the hike.

**Areas of controversy:**

There is no consensus on how to standardize trail recommendations to account for individual variability.

**Growing points:**

Personalized hiking advice integrating individual fitness, cardiovascular risk, and trail features may enhance safety. Wearable technologies enable real-time adjustment of exertion levels.

**Areas for developing research:**

New tools combining personal health data and environmental features to optimize hiking safety and accessibility should be implemented.

## Introduction

Mountain tourism is a continually growing sector, attracting >400 million visitors to mountain regions annually [1] Hiking, defined as the activity of walking in a mountainous environment, mainly on marked trails with the aim of reaching a point of interest, is the most practised outdoor activity in the mountains [2] Hiking offers numerous health benefits, ranging from immediate effects such as reduced blood pressure, decreased stress levels, improved immune system functioning, to long-term outcomes including weight loss, reduced subclinical inflammation, decreased depression rates, and overall well-being [3–6] This outdoor activity can require considerable physical effort and cardiorespiratory demands depending on trail characteristics as well as environmental factors, including changes in temperature, humidity, and partial oxygen pressure. Such conditions lead to (patho-)physiological responses and adaptations, predominantly within the cardiopulmonary system, to ensure adequate peripheral tissue oxygenation [7].

Although most hiking excursions are well tolerated and incident-free, this outdoor activity ranks first in requiring rescue operations, amounting to ~50% of all rescue calls [8]. The predominant causes for intervention are falls/slips, followed by physical inability to continue, often due to underestimation of the physical effort required for a given trail [9]. While most hiking accidents are nonfatal, sudden cardiac death represents the leading cause of death among males over 34 years of age during excursions [10].

Mountain signage and dedicated websites try to prevent such adverse events and provide useful preparative information for hikers including average estimated hiking time as well as trail characteristics and difficulty. Despite this, there is usually no information available regarding the required physical effort, and when present, this is generic, not suitable for everyone. The purpose of this study is to objectively investigate the required exercise intensity needed for hiking a trail classified as ‘easy’ by mountain signage and to understand which individual factors and trail/environment characteristics may affect the associated cardiorespiratory and metabolic response.

## Methods

### Study design

This study was designed as a cross-sectional observational analysis aimed at exploring the physiological effort during hiking, using a novel integration of laboratory and field-based testing, consisting of two monitored exercise testing sessions performed within 1 week, using a portable breath-by-breath gas-analysing device. The first evaluation was conducted in an outpatient setting at the Sports and Exercise Medicine Division of the University of Padova, while the second specifically analysed an outdoor hiking activity. The recruitment of participants was carried out between May and October 2023. Participation in the study was on a voluntary basis, promoted through public posting in hospital facilities. Health status and medication use were recorded during initial screening. Inclusion criteria were an age between 18 and 69 years and the absence of any medical conditions contraindicating the performance of a maximal exercise stress test and/or an outdoor exercise protocol. Moreover, participants with any acute or chronic medical conditions that contraindicate high-intensity exercise or that could significantly alter oxygen consumption (VO_2_), such as severe cardiovascular, respiratory, or musculoskeletal disorders, were excluded from the study. All participants provided written informed consent and were covered by insurance for conducting the outdoor test. This study was performed in accordance with the Declaration of Helsinki and approved by the Local Clinical Research Ethics Committee (AOP3157; CET Code:5855/AO/23).

### Outpatient evaluation

The included participants underwent an outpatient medical evaluation, comprehensive of a medical history assessment, physical examination, and anthropometric measurements. The physical activity level was recorded as minutes of moderate–vigorous physical activity per week and classified according to the World Health Organization Recommendations [11]. Blood tests including total cholesterol, High-Density Lipoprotein-Cholesterol (HDL-C), Low-Density Lipoprotein-Cholesterol (LDL-C), and triglyceride values and smoking status were carried out to estimate cardiovascular risk [12]. Cardiovascular risk was estimated using the SCORE2 algorithm, which provides 10-year risk predictions of fatal and nonfatal cardiovascular events based on age, sex, smoking status, systolic blood pressure, and non-HDL cholesterol [12].

All participants performed a maximal incremental cardiopulmonary exercise test (CPET) on a treadmill (T170 DE, h/p Cosmos, Nussdorf-Traunstein, Germany) using a standardized Bruce ramp protocol with a portable gas-analysing device (K5, Cosmed, Rome, Italy) and a heart rate (HR)–monitoring chest strap (HRM-Dual, Garmin, USA). Criteria of exhaustion were defined as a Borg rating of perceived exertion ≥18/20 associated with a respiratory exchange ratio (RER) > 1.10. Peak oxygen consumption (VO_2_ peak) was defined as the average value of VO_2_ within the 30-s interval before peak exercise [13]. All tests were conducted under the supervision of a Sports and Exercise Medicine physician. Blood pressure was measured at rest, at peak, and during the recovery phase [14]. Ventilatory and gas exchange measurements were sampled breath-by-breath. The first ventilatory threshold (VT1) was determined using the V-slope method and integrating it with the analysis of the ventilatory equivalents for oxygen. The second ventilatory threshold (VT2) was identified by an increase in the ventilatory equivalent for carbon dioxide and a decrease in end-tidal pressure of carbon dioxide [15].

### Outdoor evaluation

**Figure 1:**
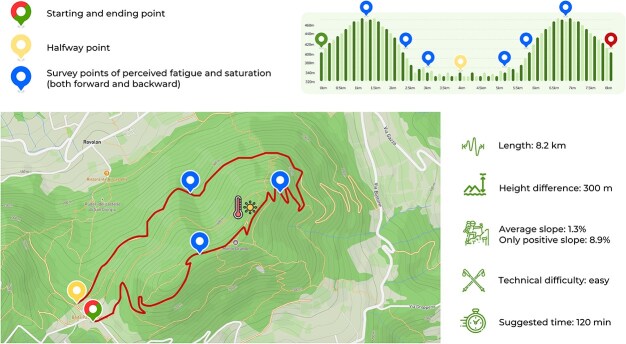
Map of the selected trail. Outdoor testing took place during the mornings between June and October 2023. The trail was hiked in the counterclockwise direction (from green to yellow placemark) and then in the clockwise direction (from yellow to red placemark). Additional assessments of rating of perceived exertion and saturation were made at the points indicated by the blue placemarks. The thermometer icon indicates an official weather station whose measurements were compared with those registered by the portable gas analyser.

Within 1 week of the outpatient evaluation, all participants underwent the outdoor hiking testing. All hiking tests were performed between 8:00 a.m. and 2:00 p.m. under dry, stable weather conditions. The trail selected for these tests was the ‘Monte Grande Trail’ in the Colli Euganei Regional Park of the Veneto Region in Italy (Fig. 1), which is classified as ‘easy’ by mountain signage. The trail is 8.2 km long and has an elevation gain of 300 m. The trail circuit was hiked by each subject consecutively in both directions (outward and return). All subjects wore the same portable gas analysis CPET device and a chest strap for HR monitoring. The gas analyser was equipped with Global Positioning System (GPS) tracking. All equipment was sanitized prior to each use, following manufacturer and institutional hygiene protocols, and the portable gas analyser was calibrated before every session and for each subject to ensure reliability of gas exchange measurements. Each participant received standardized pretest instructions, including guidance on preparation, hydration, and nutrition. They were instructed to avoid strenuous activity in the 24 h preceding the test, maintain regular sleep routines, and consume a light standardized breakfast at least 1 h before starting the hike. Each participant was asked to complete the trail simulating as much as possible a real and independent hiking activity, maintaining a self-selected but comfortable pace consistent with recreational hiking behaviour. No running, stopping, or talking was allowed during the hike, except in cases of absolute necessity. No drinking or eating was provided during the hike. All participants were weighed via a digital bioimpedance scale before and after the test. All tests were conducted under the supervision of a Sports and Exercise Medicine physician and a graduate in Sports Science, who did not interfere with the participants’ walk following the subject at a distance of ~30 m. During the trail, ratings of perceived exertion (RPE-Borg 6–20), as well as peripheral oxygen saturation, were recorded at specific control points. Ventilatory and gas exchange measurements were sampled breath-by-breath, determining exercise intensities using the VTs, as recently proposed by our group [16]. VT1 marks the limit between light to moderate intensity, while VT2 marks the limit between moderate to high intensity effort. Environmental parameters have also been registered: altitude, slope, barometric pressure, wind speed, temperature, and relative humidity. The portable gas analyser has a built-in barometer in addition to four built-in temperature and humidity sensors. The data collected by the device were compared on a daily basis with those emitted by an official weather station located along the trail. A visual representation of selected moments from the outpatient and outdoor testing procedures is provided in Supplementary Fig. S1.

### Statistical analyses

The Shapiro–Wilk test was used to assess for normal distribution of all parameters. Continuous variables were expressed as mean ± standard deviation or median (interquartile range), and comparisons between subgroups were performed using the Student’s *t*-test for independent samples or the Mann–Whitney test for variables with normal and non-normal distributions, respectively. Categorical variables were expressed as frequencies (percentages) and compared between groups using Pearson’s chi-square test. The bivariate correlations were evaluated with Pearson’s or Spearman’s correlation index if they were normally or non-normally distributed, respectively. No formal sample size calculation was performed due to the exploratory nature of the study.

Relative oxygen consumption during a hike compared to VO_2_ peak at CPET was defined as VO_2_-hike. HR-hike and VE-hike have been defined using the same method. The effects of individual and environmental characteristics on required exercise intensity to complete the trail were determined by two multivariate analyses: individual characteristics: a linear multiple regression analysis was performed to assess the individual hiker’s determinants of the VO_2_-hike; and environmental characteristics: the sampled VO_2_ values were averaged across participants considering the time elapsed between 10 breaths along the trail. Subsequently, a linear multiple regression analysis was performed to assess environmental determinants for exercise intensity. All reported probability values are two-tailed, and a *P*-value < .05 was considered statistically significant. The OMNIA software was used to elaborate CPET data, while the SPSS software version 26 was used for the data analyses.

## Results

### Outpatient and outdoor evaluations

During the study period, 72 subjects (65% men) were evaluated. The average age of the included participants was 43.53 ± 14.99 years. The main demographic and clinical characteristics, as well as the outpatient CPET data of the study population, are summarized in Table 1. Eighteen participants (25%) had at least one chronic condition: arterial hypertension (*n* = 7), dyslipidaemia (*n* = 6), type 2 diabetes (*n* = 2) and type 1 diabetes (*n* = 1), stable coronary artery disease (*n* = 2), multiple sclerosis (*n* = 1), amyotrophic lateral sclerosis (*n* = 1), Parkinson’s disease (*n* = 1), hypothyroidism (*n* = 1), and ulcerative recto-colitis (*n* = 1). Participants completed the outpatient test until exhaustion without any cardiorespiratory symptoms; all tests were maximal in terms of perceived exercise intensity (18.5/20 ± 0.7 RPE) and/or RER (1.14 ± 0.06).

**Table 1:** Baseline characteristics and outpatient maximal CPET evaluations and outdoor test of the included participants (*n* = 72)

Outpatient test		Outdoor test	
Clinical evaluation		Hiking test	
Age (years)	43.5 ± 14.9	Hiking time (min)	97 ± 11^a^
Gender (female%)	25 (35%)	Speed (km/h)	4.2 ± 0.5
Weight (kg)	75.2 ± 14.3	Average VO_2_ (ml/min)	1339.0 ± 479.4
Height (cm)	175.1 ± 9.1	Maximal VO_2_ (ml/min)	2386.5 ± 624.9
BMI (kg/m^2^)	24.5 ± 3.8	Average VO_2_/kg (ml/kg/min)	17.9 ± 5.5
Physical activity level		Maximal VO_2_/kg (ml/kg/min)	32.2 ± 7.0
Sedentary	4 (6%)	Average RER	0.97 ± 0.29
Low	16 (22%)	Maximal RER	2.15 ± 0.69
Moderate	27 (37%)	Average VE (l/min)	38.0 ± 11.4
High	25 (35%)	Maximal VE (l/min)	66.8 ± 18.9
Smoking history (%)	8 (11%)	Average HR (bpm)	111.0 ± 24.9
SBP rest (mmHg)	123.2 ± 11.4	Maximal HR (bpm)	145.8 ± 28.1
Total cholesterol (mg/dl)	182.3 ± 53.4	Average saturation (%)	96 ± 12
CV risk score		Average physical effort (RPE)	11.8 ± 1.8
Low-to-moderate	45 (63%)	Maximal physical effort (RPE)	14.3 ± 2.6
High	19 (26%)	Weight loss (kg)	0.71 ± 0.35
Very high	8 (11%)	Energy expenditure (kcal)	611.6 ± 200.9
Maximal CPET parameters		Environmental parameters	
VT1 (ml/min)	2036.9 ± 581.9	Temperature (C°)	20.3 ± 4.5
VT2 (ml/min)	3012.6 ± 878.9	Atmospheric pressure (mmHg)	732.1 ± 4.1
VO_2_ peak (ml/min)	3317.2 ± 951.3	Relative humidity (%)	76.2 ± 13.2
VO_2_ peak/kg (ml/kg/min)	44.7 ± 11.3	Wind speed (m/s)	2.5 ± 1.0
VE peak (l/min)	98.8 ± 28.9	Average altitude (m)	398.8 ± 48.9
HR peak (bpm)	169.7 ± 22.2	Height difference (m)	300
SBP peak (mmHg)	179.1 ± 22.3	Average positive slope (%)	8.9
Exercise intensity during hike			
Average VO_2_-hike (%)	41.50 ± 14.28	Maximal VO_2_-hike (%)	74.05 ± 16.21
Average VE-hike (%)	41.09 ± 16.30	Maximal VE-hike (%)	71.72 ± 24.83
Average HR-hike (%)	65.25 ± 12.40	Maximal HR-hike (%)	85.88 ± 14.46

Data are expressed as continuous variable (mean standard ± deviation) or frequencies (percentage).

BMI, body mass index; CPET, cardiopulmonary exercise testing; CV, cardiovascular; HR, heart rate; MVPA, moderate–vigorous physical activity; RER, respiratory exchange ratio; RPE, rate of perceived exertion; SBP, systolic blood pressure; VT, ventilatory threshold; VO_2_ peak, oxygen consumption at peak exercise; VE, ventilation. HR-hike = HR during hiking as % of CPET HR peak; VE-hike = VE during hiking as % of CPET VE peak; VO_2_-hike = VO_2_ during hiking as % of CPET VO_2_peak.

^a^For the two participants who completed only half of the trail, their hiking times were considered as doubled.

The physical activity level was calculated as low, moderate, or high depending on the weekly volume of physical activity practised: low: <150 min at moderate intensity, <75 min at vigorous intensity, or < at a combination of the two. Moderate: between 150 and 300 min at moderate intensity, between 75 and 150 min at vigorous intensity, or at a combination of the two. High: >300 min at moderate intensity, >150 min at vigorous intensity, or > at a combination of the two.

Two participants interrupted the outdoor test due to exhaustion, performing only the first half of the trail in the absence of cardiorespiratory symptoms. Four subjects referred slight knee pain during downhill sections without impeding the completion of the trail. The outdoor test data including CPET parameters and environmental conditions are summarized in Table 1 (total sampling measurement 114 h and 51 min). The average hiking time was 97 ± 11 min. The mean Borg rating of perceived exertion was 11.77 ± 1.75. No patients exhibited exercise-induced desaturation. The average VO_2_ measured during the trail was 1339 ± 479 ml/min (17.9 ± 5.5 ml/kg/min) with an average HR of 111 ± 25 bpm.

### Exercise intensity

The exercise intensity parameters during the outdoor test in relation to the outpatient CPET evaluations are shown in Table 1. The average VO_2_-hike, defined as the oxygen consumption during the hike expressed as a percentage of laboratory-determined VO_2_ peak, was 41.50 ± 14.28% with an average maximal value of 74.05 ± 16.21%. The rating of average and maximal perceived exertion during the hike was 11.77 ± 1.75 and 14.0 ± 1.75 according to the Borg scale, respectively. The two subjects who stopped the outdoor activity prematurely reported a Borg rating of 18–19/20.

**Figure 2:**
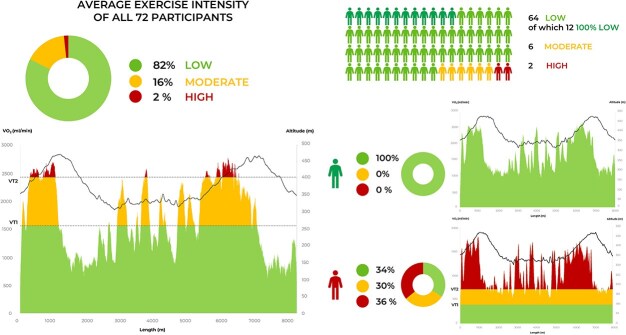
Exercise intensity between participants. The left side shows the average exercise intensity of all participants during the hiking trail. The right side shows the highest registered exercise intensity component among all participants and two selected explanatory cases. Exercise intensity zones were defined individually based on ventilatory thresholds: low (green) = below VT1, moderate (yellow) = between VT1 and VT2, high (red) = above VT2.

On average, 82.3 ± 16.5% of the trail was conducted at low intensity, 16.0 ± 11.5% at moderate intensity, and 1.70 ± 6.0% at high intensity. There was great heterogeneity among the participants; indeed, 17% completed the entire trail at low intensity, while two subjects spent more time at high intensity than at moderate or low intensity (Fig. 2).

### Subgroup analyses

**Figure 3:**
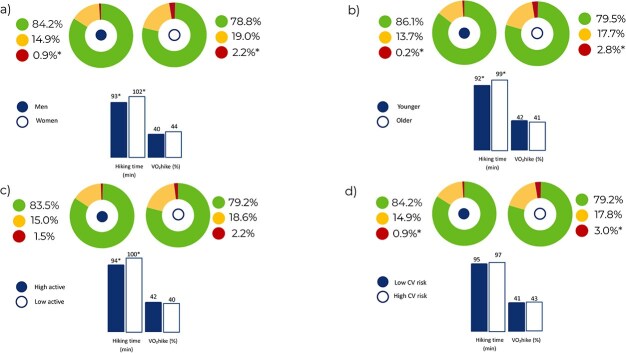
Subgroup analysis for main parameters. The groups compared are: (a) males vs females; (b) younger (<40 years old) vs older (≥40 years old); c) highly active (≥150 min/week of moderate physical activity or ≥75 min/week of vigorous physical activity) vs less active (<150 min/week of moderate physical activity or <75 min/week of vigorous physical activity); and (d) low cardiovascular risk (low-moderate SCORE2) vs high cardiovascular risk (high-very high SCORE2). The parameters represented are exercise intensity, hiking time, and VO_2_-hike. VO_2_-hike refers to relative oxygen consumption during hiking (% of VO_2_ peak). Exercise intensity zones were defined individually based on ventilatory thresholds: low (green) = below VT1, moderate (yellow) = between VT1 and VT2, high (red) = above VT2. Asterisk represents a statistically significant difference between groups. VO_2_-hike showed no difference in any subgroup analysis.

Male participants, younger subjects, and those engaging in more physical activity were able to complete this trail in less time, showing a higher average and maximal cardiorespiratory fitness. On the other hand, females, as well as older and less trained subjects showed a longer time spent in high intensities. These findings are detailed in Supplementary Table S1 for sex, Supplementary Table S2 for age, and Supplementary Table S3 for physical activity level. No difference in hiking time was observed between participants with low/moderate and high/very-high cardiovascular risk as determined by SCORE2. However, participants with high/very high SCORE2 revealed a longer time spent in high-intensity physical activity when compared with subjects at low/moderate risk (3.2% vs 0.7% of the overall hiking time; *P* < .001), as shown in Supplementary Table S4. These subgroup comparisons are also visually represented in Fig. 3.

### Individual and environmental determinants of exercise intensity

Bivariate correlation analyses revealed a significant positive association between VO_2_-hike and cardiovascular risk, as estimated by the SCORE2 algorithm (*r* = 0.225; *P* = .046). The multivariate logistic regression analysis evaluating the hikers’ biological determinants of VO_2_-hike revealed the cardiovascular risk score expressed as SCORE2 as a unique independent predictor (*B* = 1.70 ± 0.58, *P* = .005; Table 2).

**Table 2:** Multiple regression analysis for the hikers’ biological determinants of VO_2_-hike

Predictors	Beta	95% confidence interval	*P*-value
Gender (male)	6.230	−1.659—14.118	.120
Age (years)	−0.321	−0.677—0.034	.076
BMI (kg/m^2^)	0.816	−0.110—1.743	.083
Physical activity level (4 scales)	1.198	−2.780—5.177	.550
Resting blood pressure (mmHg)	−0.217	−0.554—0.121	.204
CV risk (SCORE2)	1.696	0.541–2.851	.005

R2/R2 adjusted = 0.399/0.159.

Bivariate correlation analyses between VO_2_-hike and environmental parameters revealed significant positive associations with altitude (*r* = 0.194; *P* = .003) and slope (*r* = 0.857; *P* < .001). The multivariate logistic regression analysis on the environmental determinants of increased VO_2_-hike showed the trail slope as the unique independent predictor (*B* = 34.42 ± 4.37, *P* < .001; Table 3).

**Table 3:** Multiple regression analysis for the environmental determinants of VO_2_-hike

Predictors	Beta	95% confidence interval	*P*-value
Temperature (C°)	15.033	−178.014—208.081	.729
Humidity (%)	3.787	−55.364—62.937	.923
Barometric pressure (mmHg)	28.168	−3.569—52.767	.250
Altitude (m)	3.471	−0.760—6.181	.130
Slope (%)	34.485	26.585—42.385	<.001

R2/R2 adjusted = 0.477/0.454.

## Discussion

Despite the evidence regarding the health benefits of hiking, the increase in mountain rescue calls suggests the need to study this outdoor activity and the respective population. Factors that may influence the occurrence of such adverse events should be identified so that appropriate preventive measures can be implemented. This study investigated the exercise intensity during an easy mountain hiking experience, exploring how the relative cardiorespiratory and metabolic effort varies among subjects. Despite the trail being labelled as ‘easy’ by mountain signage, our findings revealed a significant variability in exercise intensity and hiking time across the participants, challenging the generic estimations and recommendations provided by mountain signage and related web information. Moreover, this study provides novel insights into the mismatch between trail signage and individual cardiorespiratory effort required during hiking. Study outcomes suggest that by linking individual fitness and cardiovascular risk factors to real-time exertion levels, this may allow redefining hiking recommendations through a more personalized and data-driven lens [17].

### Individual factors

Sudden cardiac death is the leading cause of nontraumatic deaths during mountain hiking [18]. Since this increases sharply above 40 years of age, it is important to consider the main risk factors for cardiovascular events during moderate- and high-intensity physical activity, namely, male sex, older age, dyslipidaemia, pre-existing diseases, and low cardiorespiratory fitness [10, 19]. Indeed, subgroup analyses confirmed how performance and physiological responses to exercise were influenced by sex, age, and physical activity level. Notably, exercise intensity was found to be influenced primarily by age, physical activity level, and cardiovascular risk score, with subjects having a high-to-very-high cardiovascular risk score experiencing higher perceived exertion and engaging in exercise of a more vigorous intensity for a potentially dangerous period >3% of the overall hiking duration. For a hike of ~2 h, comparable to the assessed outdoor activity, the average time spent at high intensity corresponds to ~4 min, but for longer hikes, it can reach several minutes or hours, thus also affecting recovery. This could explain the relevant percentage of subjects calling for rescue service due to physical inability during hiking [8]. Indeed, the two participants who did not complete the outdoor evaluations due to exhaustion were both at very high cardiovascular risk (both affected by stable coronary artery disease, one by diabetes) but on regular drug therapy and follow-up. This discrepancy suggests a need for more personalized exercise recommendations to ensure safety and efficacy in hiking activities, especially in higher-risk individuals. Current guidelines highlight that individuals with high or very high cardiovascular risk score should undergo maximal exercise testing before performing vigorous intensity physical activity [20], and this study shows that even for easy-classified trails at low altitude, some sections may still require vigorous or near maximal intensity.

### Environmental factors

One of the primary environmental factors influencing exercise intensity during hiking is the slope of the trail. Uphill sections clearly demand greater energy expenditure and VO_2_ due to the increased muscular effort required to overcome gravity [21]. Slope has been quantitatively shown to be the most critical environmental determinant of exercise intensity during uphill walking [22, 23]. An average ascent rate of 300 m per hour (up to an altitude of 3500 m) could require an altitude-adjusted relative VO_2_-hike of 18–22 ml/min/kg, which corresponds to different relative exercise intensity ranges depending on the individual hiker’s conditions and fitness [17].

While this metabolic demand may appear to be a moderate effort for some subjects, it can pose significant cardiorespiratory strain for individuals with reduced functional capacity. Therefore, realistic assessments of individual fitness and aerobic performance are advisable preventive measures for mountain activities, especially among elderly or chronically ill participants [17].

While other factors like altitude, temperature, and humidity may also affect exercise capacity, their impact was less important in our study setting due to limited hiking duration, stable environmental conditions, and low altitude. Indeed, high altitudes reduce the oxygen availability due to hypobarism, increasing the difficulty of aerobic physical activities and necessitating greater cardiovascular and ventilatory effort [24]. Similarly, extreme temperatures and high humidity can alter physiological responses to exercise, affecting thermoregulation and fluid balance, thus influencing VO_2_ [25]. However, in our study, due to the minimal variations in these environmental conditions across the evaluated trail, significant differences in VO_2_-hike attributable to these factors were not observed, but they should be considered in specific circumstances.

### Possible strategies

It is thus needed to find strategies for adapting mountain-hiking recommendations considering individual and environmental characteristics to facilitate also patients’ engagement in beneficial and safe outdoor physical activities [26]. Similar approaches have been proposed in other outdoor sports, such as sport climbing, where specific functional assessments are used to guide individualized performance recommendations [27]. Subjective intensity monitoring could be proposed for hiking, but a previous study has shown that the Borg rating of perceived exertion during uphill walking is generally higher on a treadmill than during outdoor activity [28]. This suggests that RPE should be interpreted with caution in nonlaboratory conditions, where environmental and psychological factors may influence subjective perception of effort. For this reason, an objective measurement of exercise intensity can help to improve safety and may help respect exercise intensities during hiking. Currently, not being feasible to sample VO_2_ during hiking, HR monitoring can be an excellent surrogate for exercise intensity and it has become largely accessible with devices such as smartwatches/band or chest straps. Thus, the hikers, knowing their HR intensity thresholds based on clinical evaluations or exercise testing, can control their exercise intensity during the outdoor activity. Our group recently proposed another possible solution with the creation of a tool to estimate the feasibility of a trail, based on individual characteristics of the hiker and the selected trail [29]. Combining the user’s biological information, including health status, and the trail information (i.e. GPS track), this tool provides personalized guidance on the recommended hiking time (i.e. as a surrogate for exercise intensity), required physical effort, and other useful information, including tips and precautionary measures for populations with chronic diseases. This digital tool showed that its estimated hiking times are closer approximations to actual performance when compared to existing methods such as trail signage [29].

Personalized planning can be particularly helpful for individuals with chronic conditions, as cardiovascular events may be triggered by unusual physical exertion, especially in older adults or those with low physical fitness [8, 9, 29, 30].

These issues, perspectives, and related study outcomes emphasize the need to develop new approaches or tailored recommendations for mountain hiking in order to mitigate the risk of adverse events. The interindividual variability shown for exercise intensity during hiking, influenced by personal and environmental characteristics, indicates that a one-size-fits-all approach may not be adequate for ensuring safety and maximizing health benefits during physical activity in the mountains.

Future research should explore strategies to integrate mountain signage with more individualized evaluations, including the subject’s health status, physical fitness, and trail-specific environmental conditions, while also considering personal preferences, just like with tailored exercise prescription [31]. In this context, pre-hiking assessments, ranging from risk stratification tools to simplified fitness measurements or maximal CPET, may help identify individuals more likely to exceed safe intensity thresholds, particularly in mountains. Additionally, wearable technologies and mobile health applications could enable real-time monitoring of physiological and environmental parameters (e.g. HR, exercise intensity, GPS-tracked elevation gain, or critical slopes), offering personalized feedback and supporting adaptive pacing throughout the hike, potentially reducing the risk of overexertion and enhancing both safety and user confidence during hiking [28, 29, 32].

Moreover, educational programmes that address technical difficulty, activity planning, and environmental conditions, combined with structured training programmes have been shown to improve both safety and perceived control during mountain activities [10, 30, 33].

Such strategies may be especially valuable in individuals with known or potential cardiovascular risk, for whom overexertion or misjudgement of trail difficulty could have serious consequences. However, beyond individual-level interventions, broader public health efforts should aim to improve trail signage clarity, increase user awareness regarding physical demands, and promote the strategic placement of automated external defibrillators in high-frequency hiking areas to mitigate risk and improve emergency procedures [25, 33–35].

### Limitations and perspectives

This study presents several strengths, including the combination of outpatient CPET with outdoor breath-by-breath cardiorespiratory analysis during a long-term hiking activity conducted on real hiking trails, leading also to clinical and public health implications with concrete preventive measure proposals. However, some limitations must be acknowledged. First, the study was designed as a cross-sectional, exploratory investigation and was not powered to establish causal relationships. While the sample size is modest, it is consistent with prior field-based physiological studies and allowed meaningful subgroup comparisons and multivariate regression analyses. The need to conduct the outdoor activity within one week of the outpatient CPET, coupled with the technical challenges of outdoor testing (no more than two tests per day) and the dependence on weather conditions (tests were not conducted on rainy days or in wet soil conditions), limited the increase in sample size. Moreover, the relatively healthy, physically active profile of the participants may limit the generalizability of our findings to more sedentary or clinically vulnerable populations. Furthermore, although test protocol, related procedures, and environmental conditions were standardized as possible to minimize confounding, the influence of unmeasured factors such as sleep quality, nutrition, emotional stress, and pacing strategies cannot be entirely excluded. Also, CPET results in a controlled outpatient setting may not fully replicate the physiological demands experienced during real outdoor hiking [36]. It should also be considered that the environmental conditions of the selected trail were relatively constant and may not reflect the diversity of conditions encountered in different hiking locations, which may limit the applicability of the findings to other trails with varying environmental challenges. Moreover, the restriction of food and fluid intake and the use of a mask during the outdoor evaluation might also affect performance and perceived exertion. Future research should aim to include broader demographic and clinical profiles and vary trail conditions, in order to expand outcomes and subsequently adapt implementation strategies of the findings. Nonetheless, we consider this balance between experimental control and outdoor validity to enhance the translational relevance of our findings, providing meaningful insights into the physiological demands of hiking as experienced in real-world conditions.

## Conclusion

A significant variability in hiking time and exercise intensity has been found during the same easy-labelled trail, with parts at high intensities potentially unsafe for people with high cardiovascular risk. Trail and individual characteristics, particularly slopes and cardiovascular risk, influence the time spent at different exercise intensities. Therefore, solutions are needed to provide more individualized guidance to hikers, especially for those with high cardiovascular risk.

## Supplementary Material

Supplementary_material_1_idaf019

## Data Availability

The data that support the findings of this study are available from the corresponding author upon reasonable request.
