# Supplementary material for: Are hiking recommendations one-size-fits-all? Insights into cardiovascular safety and trail demands
Source: Br Med Bull. 2025 Nov 19;156(1):ldaf019. doi: 10.1093/bmb/ldaf019 (PMC12698341; doi:10.1093/bmb/ldaf019)
Supplement: Supplementary_material_1_idaf019 [file supplementary_material_1_idaf019.pdf]

Supplementary Figure 1. Visual documentation of the laboratory and outdoor testing protocol.

This composite panel illustrates representative phases of the cardiopulmonary assessment protocol described in the manuscript.
Top left: Laboratory setup for resting gas analysis before CPET.
Top center-left: Participant performing maximal CPET with K5 metabolic system and gas mask on a treadmill.
Top center-right: Real-time display of physiological variables during field assessment (e.g., VO_2, ventilation).
Top right: Calibration of the gas analyzer prior to testing.
Bottom left and bottom center-left: Participant equipped with the portable K5 system at the beginning and the end of the hiking trail, near trailhead signage.
Bottom center-right: Borg Rating of Perceived Exertion (RPE) scale used to record subjective exertion at predefined checkpoints.
Bottom right: A participant is reached by two staff members (a physician and graduate in Sports Science) at a control point to record peripheral oxygen saturation and perceived exertion using the Borg scale.

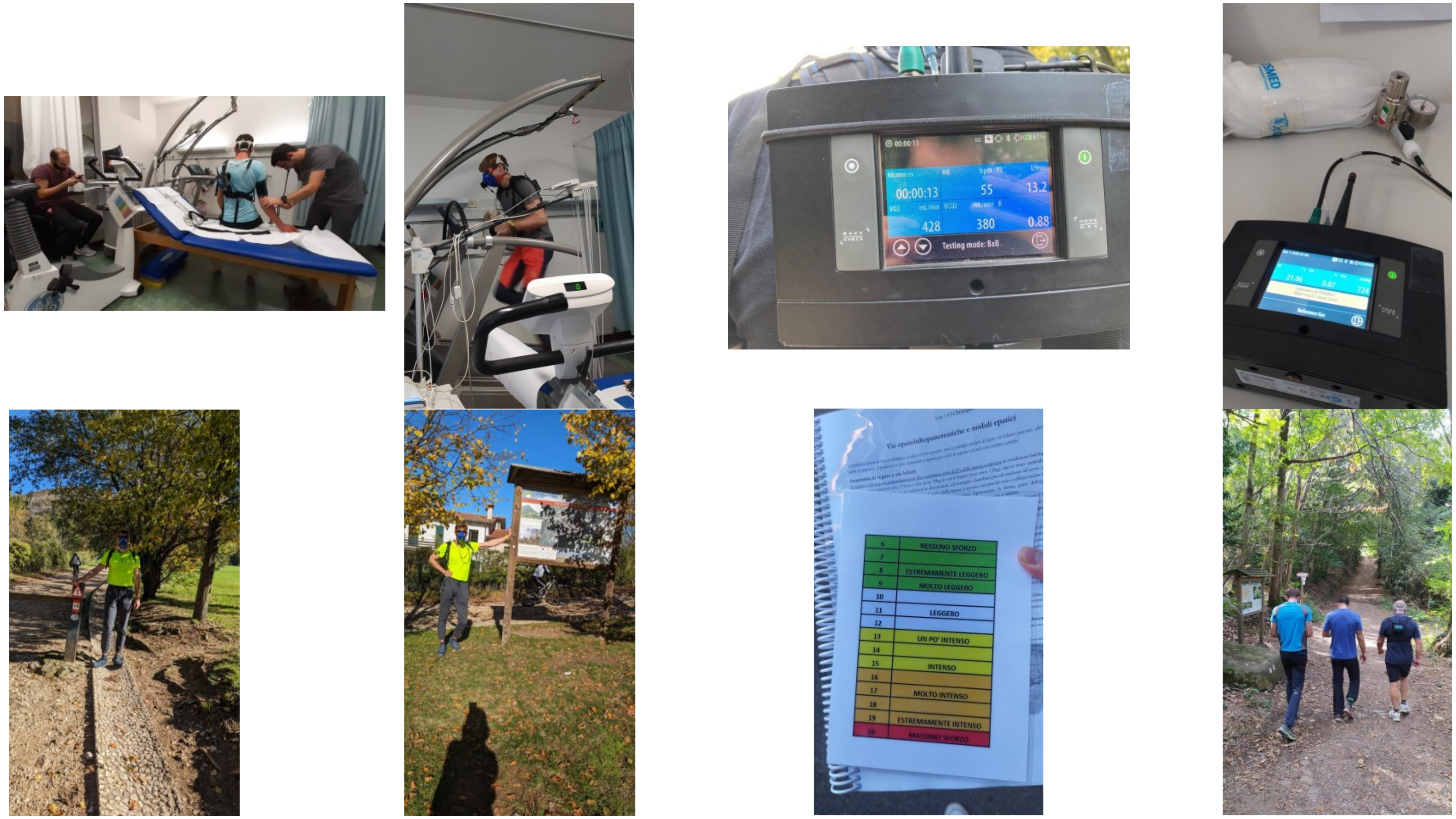

Table S1. Subgroup analysis of outdoor tests divided by gender.

	Men (n=47)		Women (n=25)		
	Mean	SD	Mean	SD	P
Age (years)	42.65	14.63	45.2	15.8	0.496
BMI (kg/m^2)	25.38	4.03	22.72	2.61	0.004
Hiking time (min)	92.56	12.23	101.63	12.87	0.004
VE average (L/min)	41.57	11.77	31.21	6.86	<0.001
VE max (L/min)	74.12	18.3	53.13	10.89	<0.001
VO_2 average (ml/min)	1492.45	486.47	1050.58	305.61	<0.001
VO_2 max (ml/min)	2668.6	546.58	1856.09	365.53	<0.001
VCO_2 average (ml/min)	1348.27	401.76	952.87	235.81	<0.001
VCO_2 max (ml/min)	2520.72	558.62	1757.88	324.68	<0.001
RER average	0.96	0.28	0.99	0.31	0.683
VO_2/kg average (ml/min/kg)	18.61	5.84	16.86	4.82	0.114
VO_2/kg max (ml/min/kg)	33.37	7.27	29.88	5.99	0.057
MET average	5.21	1.71	4.82	1.38	0.330
MET max	9.53	2.08	8.54	1.72	0.046
HR average (bpm)	114.55	21.65	104.56	29.38	0.123
HR max (bpm)	147.12	25.3	143.26	33.0	0.600
Energy expenditure (kcal)	661.87	207.83	517	149.63	0.003
Speed average (km/h)	4.37	0.48	3.95	0.42	<0.001
Speed max (km/h)	6.11	0.71	5.64	0.53	0.005
Perceived exertion average (RPE)	11.72	1.55	11.87	2.12	0.720
Perceived exertion max (RPE)	14.35	1.74	14.26	2.20	0.880
HR hike (%)	65.52%	11.48%	64.75%	14.15%	0.940
HR hike max (%)	84.07%	13.50%	89.06%	15.85%	0.058
VO_2 hike (%)	40.00%	13.85%	43.83%	14.69%	0.230
VO_2 hike max (%)	72.29%	15.48%	77.38%	17.36%	0.123
Light intensity (%)	84.19%	16.45%	78.82%	16.43%	0.129
Moderate intensity (%)	14.88%	14.71%	19.02%	15.51%	0.213
High intensity (%)	0.93%	6.13%	2.16%	5.83%	0.043

Data are expressed as continuous variable (mean standard ± deviation). The values defined as *average* represent the mean values of the entire outdoor tests while the values defined as *max* represent the mean of the maximum value achieved during the outdoor test by every participant.

BMI: body mass index; HR: heart rate; RER: respiratory exchange ratio; RPE: rate perceived exertion; VT: ventilatory threshold; VCO_2: carbon dioxide production; VO_2: oxygen consumption; VE: ventilation.

Table S2. Subgroup analysis of outdoor tests divided by age.

	Younger; < 40 years (n=31)		Older; > 40 anni (n=41)		
	Mean	SD	Mean	SD	P
Age (years)	28.06	3.61	55.23	7.95	<0.001
BMI (kg/m^2)	24.01	4.28	24.79	3.40	0.388
Hiking time (min)	91.56	9.03	98.85	15.85	0.019
VE average (L/min)	39.20	8.87	37.04	13.05	0.431
VE max (L/min)	69.26	15.86	65.00	20.96	0.348
VO_2 average (ml/min)	1515.57	394.55	1205.54	498.67	0.006
VO_2 max (ml/min)	2654.27	544.46	2184.00	610.85	0.001
VCO_2 average (ml/min)	1322.37	360.82	1126.76	410.17	0.039
VCO_2 max (ml/min)	2438.14	543.07	2118.01	626.98	0.026
RER average	0.88	0.08	1.05	0.36	0.017
VO_2/kg average (ml/min/kg)	20.31	3.83	16.26	6.01	<0.001
VO_2/kg max (ml/min/kg)	35.59	4.05	29.56	7.68	<0.001
MET average	5.64	1.25	4.64	1.72	0.008
MET max	10.16	1.15	8.44	2.19	<0.001
HR average (bpm)	124.22	20.08	101.64	23.91	<0.001
HR max (bpm)	159.00	22.31	136.34	28.18	0.001
Energy expenditure (kcal)	666.26	166.11	570.22	216.57	0.044
Speed average (km/h)	4.44	0.51	4.06	0.42	0.001
Speed max (km/h)	6.22	0.69	5.75	0.62	0.003
Perceived exertion average (RPE)	11.85	1.38	11.72	1.99	0.700
Perceived exertion max (RPE)	14.37	1.94	14.28	1.89	0.630
HR hike (%)	67.73%	9.93%	63.63%	13.65%	0.345
HR hike max (%)	86.51%	11.63%	85.46%	16.18%	0.990
VO_2 hike (%)	41.77%	8.88%	41.30%	17.00%	0.968
VO_2 hike max (%)	73.08%	10.79%	74.79%	19.45%	0.637
Light intensity (%)	86.09%	13.17%	79.48%	18.32%	0.284
Moderate intensity (%)	13.68%	13.32%	17.71%	16.14%	0.561
High intensity (%)	0.23%	1.06%	2.81%	7.77%	0.024

Data are expressed as continuous variable (mean standard ± deviation). The values defined as *average* represent the mean values of the entire outdoor tests while the values defined as *max* represent the mean of the maximum value achieved during the outdoor test by every participant.

BMI: body mass index; HR: heart rate; RER: respiratory exchange ratio; RPE: rate perceived exertion; VT: ventilatory threshold; VCO_2: carbon dioxide production; VO_2: oxygen consumption; VE: ventilation.

Table S3. Subgroup analysis of outdoor tests divided by physical activity level.

	Sedentary/Low active (n=20)		Active/High active (n=54)		
	**Mean**	**SD**	**Mean**	**SD**	**P**
**Age (years)**	51.80	14.56	40.35	14.02	0.002
**BMI (kg/m^2)**	26.14	3.87	23.81	3.60	0.015
**Hiking time (min)**	100.07	14.02	93.78	12.35	0.045
**VE average (L/min)**	35.51	12.74	38.91	10.85	0.260
**VE max (L/min)**	63.83	18.47	67.98	19.16	0.408
**VO_2 average (ml/min)**	1160.43	575.35	1407.71	423.36	0.049
**VO_2 max (ml/min)**	2138.28	723.32	2481.94	561.45	0.036
**VCO_2 average (ml/min)**	1084.54	420.28	1259.61	383.79	0.096
**VCO_2 max (ml/min)**	2053.06	595.90	2333.84	602.21	0.080
**RER average**	1.11	0.41	0.92	0.20	0.068
**VO_2/kg average (ml/min/kg)**	14.74	6.18	19.25	4.76	0.005
**VO_2/kg max (ml/min/kg)**	27.32	6.85	34.02	6.19	<0.001
**MET average**	4.21	1.76	5.40	1.42	0.004
**MET max**	7.80	1.95	9.72	1.77	<0.001
**HR average (bpm)**	107.00	28.84	112.56	23.38	0.425
**HR max (bpm)**	139.44	37.07	148.17	23.81	0.265
**Energy expenditure (kcal)**	560.40	263.04	631.25	170.32	0.182
**Speed average (km/h)**	4.05	0.43	4.29	0.51	0.071
**Speed max (km/h)**	5.59	0.61	6.09	0.67	0.005
**Perceived exertion average (RPE)**	12.56	2.15	11.48	1.50	0.070
**Perceived exertion max (RPE)**	14.89	2.00	14.10	1.84	0.26
**HR hike (%)**	65.44%	14.45%	65.19%	11.89%	0.681
**HR hike max (%)**	84.98%	19.18%	86.14%	12.99%	0.490
**VO_2 hike (%)**	40.08%	17.34%	42.05%	12.65%	0.890
**VO_2 hike max (%)**	74.42%	18.84%	73.91%	15.30%	0.624
**Light intensity (%)**	79.21%	18.79%	83.52%	15.60%	0.512
**Moderate intensity (%)**	18.63%	15.62%	14.95%	14.82%	0.465
**High intensity (%)**	2.15%	7.74%	1.52%	5.27%	0.397

Data are expressed as continuous variable (mean standard ± deviation). The values defined as *average* represent the mean values of the entire outdoor tests while the values defined as *max* represent the mean of the maximum value achieved during the outdoor test by every participant.

BMI: body mass index; HR: heart rate; RER: respiratory exchange ratio; RPE: rate perceived exertion; VT: ventilatory threshold; VCO_2: carbon dioxide production; VO_2: oxygen consumption; VE: ventilation.

Table S4. Subgroup analysis of outdoor tests divided by cardiovascular risk score.

	Low-Moderate CV risk score (n=45)		High-Very High CV risk score (n=27)		
	Mean	SD	Mean	SD	P
**Age (years)**	35.57	12.05	56.81	8.62	<0.001
**BMI (kg/m^2)**	23.82	3.91	25.51	3.42	0.016
**Hiking time (min)**	95.05	11.40	96.80	15.72	0.585
**VE average (L/min)**	36.47	9.29	40.48	14.11	0.150
**VE max (L/min)**	63.96	16.30	71.62	22.16	0.097
**VO_2 average (ml/min)**	1346.63	449.33	1326.35	534.34	0.864
**VO_2 max (ml/min)**	2404.80	632.74	2355.93	622.24	0.750
**VCO_2 average (ml/min)**	1206.15	372.90	1219.04	446.76	0.896
**VCO_2 max (ml/min)**	2240.41	573.63	2281.56	675.79	0.784
**RER average**	0.94	0.23	1.03	0.36	0.812
**VO_2/kg average (ml/min/kg)**	18.63	4.96	16.94	6.34	0.141
**VO_2/kg max (ml/min/kg)**	33.36	6.20	30.14	7.92	0.002
**MET average**	5.21	1.47	4.84	1.81	0.348
**MET max**	9.53	1.77	8.61	2.26	0.059
**HR average (bpm)**	112.33	26.94	108.78	21.32	0.584
**HR max (bpm)**	148.68	30.41	140.75	23.30	0.275
**Energy expenditure (kcal)**	612.51	183.97	610.00	230.16	0.959
**Speed average (km/h)**	4.28	0.55	4.13	0.38	0.239
**Speed max (km/h)**	6.01	0.70	5.85	0.67	0.341
**Perceived exertion average (RPE)**	11.46	1.89	12.28	1.40	0.040
**Perceived exertion max (RPE)**	14.07	2.20	14.72	1.21	0.320
**HR hike (%)**	63.58%	12.34%	67.81%	12.30%	0.165
**HR hike max (%)**	84.62%	15.18%	87.80%	13.36%	0.47
**VO_2 hike (%)**	40.71%	12.11%	42.82%	16.88%	0.412
**VO_2 hike max (%)**	72.78%	15.53%	76.18%	17.39%	0.298
**Light intensity (%)**	84.18%	15.55%	79.24%	17.92%	0.325
**Moderate intensity (%)**	14.88%	14.82%	17.80%	15.47%	0.438
**High intensity (%)**	0.94%	4.13%	2.96%	5.19%	0.007

Data are expressed as continuous variable (mean standard ± deviation). The values defined as *average* represent the mean values of the entire outdoor tests while the values defined as *max* represent the mean of the maximum value achieved during the outdoor test by every participant.

BMI: body mass index; HR: heart rate; RER: respiratory exchange ratio; RPE: rate perceived exertion; VT: ventilatory threshold; VCO_2: carbon dioxide production; VO_2: oxygen consumption; VE: ventilation.
